# Brain glutamate in medication-free depressed patients: a proton MRS study at 7 Tesla

**DOI:** 10.1017/S0033291717003373

**Published:** 2017-12-11

**Authors:** Beata R. Godlewska, Charles Masaki, Ann L. Sharpley, Philip J. Cowen, Uzay E. Emir

**Affiliations:** 1 Department of Psychiatry, University of Oxford, Warneford Hospital, Oxford OX3 7JX, UK; 2 Oxford Centre for Functional MRI of the Brain, Nuffield Department of Clinical Neurosciences, University of Oxford, John Radcliffe Hospital, Oxford OX3 9DU, UK

**Keywords:** Basal ganglia, depression, glutamate, glutamine, MRS

## Abstract

**Background:**

The possible role of glutamate in the pathophysiology and treatment of depression is of intense current interest. Proton magnetic resonance spectroscopy (MRS) enables the detection of glutamate in the living human brain and meta-analyses of previous MRS studies in depressed patients have suggested that glutamate levels are decreased in anterior brain regions. Nevertheless, at conventional magnetic field strengths [1.5–3 Tesla (T)], it is difficult to separate glutamate from its metabolite and precursor, glutamine, with the two often being measured together as Glx. In contrast, MRS at 7 T allows clear spectral resolution of glutamate and glutamine.

**Method:**

We studied 55 un-medicated depressed patients and 50 healthy controls who underwent MRS scanning at 7 T with voxels placed in anterior cingulate cortex, occipital cortex and putamen (PUT). Neurometabolites were calculated using the unsuppressed water signal as a reference.

**Results:**

Compared with controls, depressed patients showed no significant difference in glutamate in any of the three voxels studied; however, glutamine concentrations in the patients were elevated by about 12% in the PUT (*p* < 0.001).

**Conclusions:**

The increase in glutamine in PUT is of interest in view of the postulated role of the basal ganglia in the neuropsychology of depression and is consistent with elevated activity in the descending cortical glutamatergic innervation to the PUT. The basal ganglia have rarely been the subject of MRS investigations in depressed patients and further MRS studies of these structures in depression are warranted.

## Introduction

Glutamate is the major excitatory neurotransmitter in the human brain and recent years have seen growing interest in the role of altered glutamate activity in the pathophysiology of depression and bipolar disorder (Sanacora *et al.* 2012; Taylor, 2014). This interest has been greatly stimulated by the striking antidepressant effects of the N-methyl-D-aspartate (NMDA) receptor antagonist, ketamine, in depressed patients resistant to monoamine-potentiating agents (McGirr *et al.* 2015).

It is possible to measure levels of glutamate in the living brain in an acceptable, non-invasive way, using proton magnetic resonance spectroscopy (^1^H-MRS). Several MRS studies in depressed patients have been carried out, the majority of which assessed glutamate levels in anterior brain regions such as prefrontal cortex (PFC) and anterior cingulate cortex (ACC), in comparison with those of healthy controls (Yüksel & Öngür, 2010). Generally, meta-analyses have reported diminished levels of glutamate in these regions in depression, though data from individual studies are inconsistent and a recent investigation, for example, found increased glutamate and glutamine levels in ACC in depressed patients (Luykx *et al.* 2012; Arnone *et al.* 2015; Abdallah *et al.* 2017).

One factor relevant to this inconsistency is that, at conventional field strengths [3 T (T) or less], it is difficult to separate glutamate clearly from its precursor and metabolite, glutamine, and the two compounds are usually measured together as Glx (Yüksel & Öngür, 2010; Bond & Lim, 2014). A second complicating issue is that patients have often been studied on antidepressant medication, which could alter glutamate activity (Taylor *et al.* 2008). Finally, there is the probability of clinical heterogeneity, as demonstrated by a study by Haroon *et al.* (2016) who found that glutamate levels in basal ganglia (but not ACC) were significantly higher in depressed patients with raised levels of C-reactive protein (CRP), a peripheral marker of inflammation. However, no control group was studied for comparison in this investigation.

MRS at 7 T has the spectral resolution to allow the clear identification of separate glutamate and glutamine resonances (Tkáč *et al.* 2009; Emir *et al.* 2015). The aim of the present study was to use MRS at 7 T to assess levels of glutamate and glutamine in 55 un-medicated depressed patients, and age and gender-matched controls, in three brain regions, ACC, occipital cortex (OCC) and putamen (PUT). We predicted that overall depressed patients would have lower glutamate levels than controls in ACC, but that the subgroup of patients with evidence of peripheral inflammation, as measured by CRP, would have increased glutamate and glutamine levels in PUT.

## Method

### Participants and clinical ratings

Ethical approval for the study was obtained from the National Research Ethics Service Committee (NRES), South-Central Oxford B. Fifty-five drug-free patients with an episode of major depression (31 females, 24 males, mean age 31.3 years, range 18–59 years) and 50 healthy volunteers (28 females, 22 males, mean age 31.3 years, range 19–62 years) were included in the study after giving full informed written consent. Exclusion criteria for patients included psychosis or substance dependence as defined by DSM-IV (determined using the Standard Clinical Interview for Diagnostic and Statistical Manual for Mental Health Disorders – Fourth Edition) (First *et al.* 1997), clinically significant risk of suicidal behaviour and need for urgent drug treatment; for healthy volunteers, current or past history of Axis I disorder on DSM-IV; for both groups contra-indications to magnetic resonance (MR) imaging, concurrent medication which could alter MRS neurochemicals (e.g. benzodiazepines), pregnancy or breast feeding, and involvement in a research project during the month preceding the study.

Thirty-nine patients were drug-naive and the remaining 16 patients had been drug-free for an average of 40 months (2 weeks–15 years). Of the latter group, two patients stopped medication within four weeks from the scan: one person was taking lofepramine, gabapentin and lamotrigine until two weeks before the scan, and the other was taking venlafaxine up to four weeks before the scan. Six patients had a comorbid diagnosis of generalized anxiety disorder and four suffered from occasional panic attacks, insufficient to meet criteria for panic disorder. Mood ratings were measured using the Hamilton Rating Scale for Depression (HAM-D) (Hamilton, 1960) and the Beck Depression Inventory (BDI) (Beck *et al.* 1961), while anxiety ratings were scored using the Spielberger State Anxiety Inventory (STAI) (Spielberger *et al.* 1993). We also measured anhedonia with the Snaith–Hamilton Pleasure Scale (SHAPS) (Snaith *et al.* 1995) and fatigue with the Chalder Fatigue Scale (CFS) (Chalder *et al.* 1993).

### Magnetic resonance spectroscopy

**Fig. 1.**
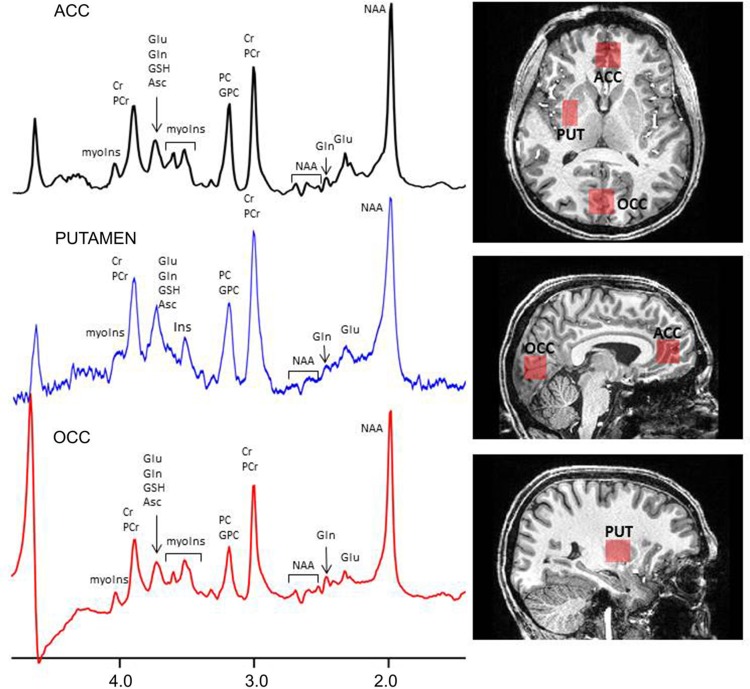
Voxel placement and representative spectra from the ACC, OCC and PUT. Glu, glutamate; Gln, glutamine; GSH, glutathione; Cr, creatine; PCr, phosphocreatine; myoIns, myo-inositol; PC, phosphocholine; GPC, glycerophosphocholine; NAA, *N*-acetylaspartate; Asc, ascorbate.

All participants had a single proton (^1^H) MRS scan at the Functional Magnetic Resonance Imaging of the Brain (FMRIB) Centre in Oxford. Scanning was performed on a 7 T Siemens MAGNETOM scanner (Siemens, Erlangen, Germany) with a Nova Medical 32 channel receive array head coil. Spectra were measured from three voxels, in the ACC (20 × 20 × 20 mm^3^), in the OCC (20 × 20 × 20 mm^3^) and in the right PUT (10 × 16 × 20 mm^3^) (Fig. 1). Voxels were positioned manually by reference to the 1 mm isotropic T1-MPRAGE image. First- and second-order shims were first adjusted by gradient-echo shimming (Shah *et al.* 2009). The second step involved only fine adjustment of first-order shims using FASTMAP (Gruetter & Tkáč, 2009). Spectra were acquired using a semi-LASER pulse sequence (TE = 32 ms, TR = 5 s, number of averages = 64) with variable power radiofrequency (RF) pulses with optimised relaxation delays (VAPOR) water suppression and outer volume saturation (Öz & Tkáč, 2011). Unsuppressed water spectra, acquired from the same voxel, were used to remove residual eddy current effects and to reconstruct the phased array spectra.

Metabolites were quantified using LCModel (Provencher, 2001). The model spectra of alanine (Ala), aspartate (Asp), ascorbate/vitamin C (Asc), glycerophosphocholine (GPC), phosphocholine (PC), creatine (Cr), phosphocreatine (PCr), *γ*-aminobutyric acid (GABA), glucose (Glc), glutamine (Gln), glutamate (Glu), glutathione (GSH), myo-inositol (myo-Ins), lactate (Lac), *N*-acetylaspartate (NAA), *N*-acetylaspartylglutamate (NAAG), phosphoethanolamine (PE), scyllo-inositol (scyllo-Ins), and taurine (Tau) were generated based on previously reported chemical shifts and coupling constants (Govindaraju *et al.* 2000; Tkáč, 2008) by using the GAMMA/PyGAMMA simulation library of VeSPA (VErsatile Simulation, Pulses and Analysis) for carrying out the density matrix formalism. Simulations were performed with the same RF pulses and sequence timings as that on the 7 T system. A macromolecule spectrum acquired from the OCC, using an inversion recovery sequence (TR  =  3 s, TE = 36 ms, inversion time TI = 0.685 s), was included in the model spectra. Metabolite concentrations were obtained relative to an unsuppressed water spectrum acquired from the same Voxel of Interest (VOI).

The MPRAGE images were segmented using SPM to determine grey matter (GM), white matter (WM), and cerebrospinal fluid (CSF) fraction (fGM, fWM, fCSF) in the voxels (Ashburner & Friston, 2005). Concentrations were then corrected for these with the following formula:
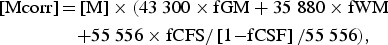
where [Mcorr] is the corrected concentration and [M] is the metabolite concentration from the LCModel output. Metabolites quantified with the Cramér–Rao lower bounds (CRLB, estimated error of the metabolite quantification) ⩾30% were classified as not detected.

### C-Reactive Protein

Venous blood samples were taken at the time of scanning and were assayed for high sensitivity (hs)CRP, using a standard immunoturbidimetric method on an Abbott c 16 000 automatic chemistry analyser (Abbott Diagnostics, Maidenhead, UK). Samples were assayed blind to diagnosis and MRS results.

### Statistics

Statistical analyses were performed in SPSS version 22. Differences in glutamate and glutamine concentrations between patients with depression and controls in the three voxels were tested using unpaired *t* tests with Bonferroni correction for multiple comparisons to yield a critical statistical *p* < 0.008. Subsequent univariate analysis of variances (ANOVAs) were used to allow for the effects of smoking, correlations were carried out using Pearson's product moment without correction for multiple analyses.

## Results

The patient and healthy control groups did not differ significantly in terms of age and gender ratio, but the body mass index (BMI) in the patients tended to be higher than controls (*t* = 1.92; *p* = 0.059). More patients than controls smoked cigarettes (18 *v.* 3; *p* = 0.001, χ^2^ test) (Table 1).

**Table 1.** Demographic data and clinical scores

	Depressed patients (55)	Healthy controls (50)
Age	31.3 (1.3)	31.3 (1.4)
Gender (F/M)	31F/24M	28F/22M
BMI	24.7 (0.7)	23.2 (0.44)
HAM-D	21.3 (0.7)	0.2 (0.1)
BDI	30.1 (1.1)	1.2 (0.2)
STAI-S	51.8 (1.6)	25.6 (0.8)
SHAPS	33.5 (0.9)	18.4 (0.6)
CFS	22.4 (0.6)	10.0 (0.4)
DSM-IV melancholia	18	N/A
Cigarette smoking	18	3

Values represent numbers or mean (s.e.m.).

HAM-D, Hamilton Rating Scale for depression; BDI, Beck Depression Inventory; STAI, Spielberger State Anxiety Inventory; SHAPS, Snaith–Hamilton Pleasure Scale; CFS, Chalder Fatigue Scale.

The following scans were rejected for reasons of quality (see Methods): in the ACC, four patients and five controls; in the OCC, seven patients and two controls; in the PUT, five patients and three controls. All remaining spectra were of acceptable quality. For results describing the signal-to-noise ratio (SNR) and full-width at half-maximum (FWHM), see Supplementary information.

**Fig. 2.**
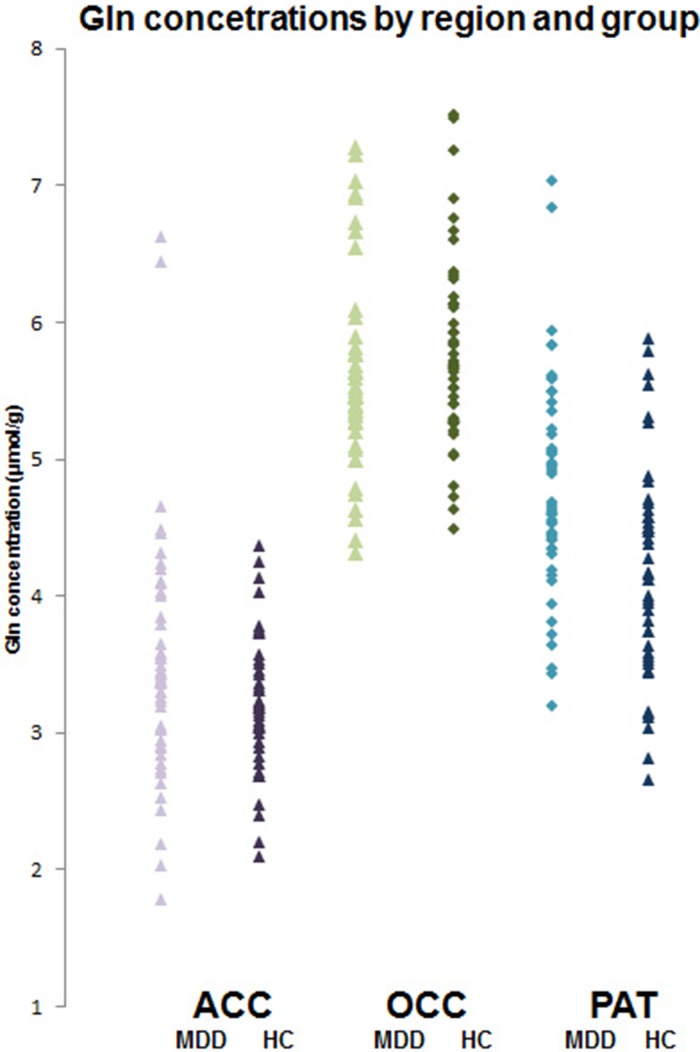
Individual glutamine concentrations (μmol/g) in depressed patients (MDD) and healthy controls (HC) in ACC, OCC and PUT. Mean glutamine level in patients in PUT is significantly higher (*t* = 3.30; *p* < 0.001, *t* test).

In ACC and OCC, there were no significant differences in concentrations of glutamate and glutamine between depressed patients and controls (Table 2). In PUT, depressed patients had significantly higher concentrations of glutamine than controls, although glutamate levels were not different (Table 2, Fig. 2). Excluding PUT scans with CRLB ⩾ 20%, gave a very similar finding (depressed patients *v.* controls, 4.91 ± 0.11 *v.* 4.38 ± 0.11 µmol/g, *t* = 3.45, *p* = 0.001).

**Table 2.** Mean (s.e.m.) absolute concentrations (μmol/g) glutamate and glutamine, corrected for GM, WM and CSF content in ACC, OCC and PUT

	Depressed patients	Healthy controls	*t* value, *p*
Glutamine (ACC)	3.48 (0.12)	3.21 (0.07)	*t* = 1.80, *p* = 0.075
Glutamine (OCC)	6.19 (0.11)	6.30 (0.12)	*t* = −0.73, *p* = 0.47
Glutamine (PUT)	4.73 (0.12)	4.17 (0.11)	*t* = 3.30; *p* < 0.001
Glutamate (ACC)	9.11 (0.14)	9.24 (0.14)	*t* = −0.66, *p* = 0.51
Glutamate (OCC)	5.61 (0.10)	5.77 (0.10)	*t* = −1.12, *p* = 0.26
Glutamate (PUT)	8.73 (0.11)	8.73 (0.13)	*t* = 0.003, *p* = 0.99

Significantly more patients than controls smoked cigarettes (Table 1) and there are reports that smoking can affect MRS glutamate measures (Durazzo *et al.* 2016). We, therefore, ran additional univariate ANOVAs on glutamate and glutamine levels in the three voxels adding ‘smoking’ as a between-subject factor to ‘diagnosis’ (depressed *v.* control). In the ACC, smoking produced a significant main effect on glutamate levels (*F* = 4.21; df = 1,92; *p* = 0.043) and there was also a main effect of diagnosis (*F* = 4.55; df = 1,92; *p* = 0.036). Mean ± s.e.m. glutamate levels were lower in the patients than controls (9.11 ± 0.11 *v.* 9.81 ± 0.29 µmol/g) and higher in smokers than non-smokers (9.79 ± 0.31 *v.* 9.13 ± 0.11 µmol/g). Similarly, in the OCC, glutamate levels were higher in smokers (*F* = 5.67; df = 1, 96; *p* = 0.019) (6.14 ± 0.22 *v.* 5.60 ± 0.22 µmol/g) but there was no main effect of diagnosis (*F* = 1.93; df = 1, 92; *p* = 0.17). There were no other main or interactive effects of smoking on glutamate or glutamine levels (all *p* > 0.05).

Eighteen depressed patients met criteria for DSM-IV depression with melancholia and had valid scan data. The MRS data of the melancholic patients were very similar to those of the non-melancholic patients and the only difference again from controls was an increased level of glutamine in the PUT (melancholic patients, 4.94 ± 0.23 µmol/g, *t* = 3.23, *p* = 0.002; non-melancholic patients 4.71 ± 0.12 µmol/g, *t* = 3.05, *p* = 0.003).

CRP levels were available for 49 patients and 44 controls. Mean CRP concentration was slightly but significantly higher in the depressed patients (0.88 ± 0.18 *v.* 0.45 ± 0.07 mg/l, *t* = 2.19, df = 61, *p* = 0.032). However, when covarying for BMI, this difference became non-significant (*F* = 1.92; *p* = 0.17). Eleven of the patients had CRP values >1.0 mg/l but there was no significant difference in glutamine levels in the PUT between these participants and patients with CRP levels <1.0 mg/l (4.44 ± 0.21 *v.* 4.86 ± 0.16 µmol/g, *t* = 1.411, *p* = 0.16). The correlation between CRP levels and PUT glutamine levels in the depressed patients was on the borderline of statistical significance but trended to a negative value (*r* = −0.31, *p* = 0.050). There were no other trends for CRP levels to correlate with neurometabolites in any of the brain regions studied (all *p* > 0.2). Similarly, allowing for BMI failed to reveal any significant correlations between CRP and neurometabolites (all *p* > 0.1).

In the patients, there were no significant correlations between scores on the HAM-D, BDI, STAI, SHAPS and CFS scales and any of the measured neurometabolites, with the exception of one uncorrected positive correlation between the total BDI score and glutamine level in ACC (*r* = 0.32, *p* = 0.025). There was generally no correlation between illness duration and MRS metabolites except for a single uncorrected positive correlation with glutamate levels in ACC (*r* = 0.36, *p* = 0.011).

## Discussion

The main finding of the study was a significant increase in glutamine levels in the PUT of patients with major depression relative to healthy controls. Although we had hypothesised reductions in MRS glutamate concentrations in ACC depressed patients relative to controls, no changes in glutamate levels were apparent in any of the three voxels examined. There was no significant correlation between CRP levels and concentrations of glutamate and glutamine in any of the voxels.

Previous meta-analytic summaries of MRS glutamate investigations in patients with unipolar depression using scanner strengths of 1.5–3 T have suggested a decrease in Glx levels in anterior brain regions in depression, particularly in ACC. However, while Luykx *et al.* (2012) (16 studies, 281 patients) found an overall lowering of both glutamate and Glx in ACC in depressed patients, Arnone *et al.* (2015) (17 studies, 363 patients) reported lower Glx but normal glutamate in prefrontal cortical regions (including the ACC). The latter group concluded that diminished Glx in depression was likely to be due to a reduction in glutamine levels, perhaps due to glial dysfunction. However, generally, sample sizes of depressed patients in the individual studies in the meta-analyses were modest (average *n* of around 20) and there was significant heterogeneity in the findings.

The use of MRS at 7 T allows a clear spectral resolution of both glutamate and glutamine (Tkáč *et al.* 2009; Emir *et al.* 2015). Of other published 7 T MRS studies in depression, Taylor *et al.* (2017) found no differences in glutamate and glutamine between 17 depressed patients and 18 healthy controls, in either ACC or thalamus, while Li *et al.* (2016) using multivoxel spectroscopic imaging (MRSI) found no significant differences in glutamate levels between 16 depressed patients and 10 healthy controls in ACC, insula, PUT, caudate and thalamus. Thus studies at 7 T, so far, have not revealed clear decreases in glutamate in ACC or other brain regions.

When allowing for the effect of smoking on glutamate levels, we did observe a lowering of glutamate levels in ACC in the depressed patients compared to controls. While this is of interest in the context of the reports noted above, the significance level of this finding was above our Bonferroni-corrected *p* value of 0.008. In addition, while we found that smoking was associated with elevated glutamate levels in ACC and OCC, previous reports of the effect of cigarette smoking on MRS glutamate in ACC have been somewhat inconsistent with increased, decreased and no change in levels being reported (Gallinat & Schubert, 2007; Mennecke *et al.* 2014; Durazzo *et al.* 2016).

The basal ganglia have been relatively little studied in MRS studies of depression despite the importance of this brain region in motor behaviour, as well as motivation, reward and emotional regulation (Gunaydin & Kreitzer, 2016). This is in part because MRS scanning of deep brain structures is challenging, especially at conventional field strengths. However, the increased SNR resulting from scanning at 7 T makes brain regions such as the PUT more feasible for MRS investigation, though the SNR of the PUT in our study was still substantially less than that of the ACC or OCC (see Supplementary data). Our finding of increased glutamine levels in the PUT was not predicted but statistically was highly significant, surviving correction for multiple comparisons. Interestingly, Haroon *et al.* (2016) found that depressed patients with evidence of peripheral inflammation, as judged by raised levels of CRP, had increased levels of glutamate in basal ganglia that correlated with measures of anhedonia.

The latter work suggests that one reason for the heterogeneity in MRS studies of glutamate in depression might be that a subgroup of patients, with evidence of inflammation, has specific disturbances in glutamate metabolism that lead to increased glutamate levels rather than the diminished glutamate concentration seen in the majority of depressed patients. It is also possible that these different profiles of glutamate abnormality in depressed patients relate more to illness course than patient subgroup, with high concentrations of glutamate in the early illness phase being followed by lower levels subsequently as a result of neurotoxic effects on glutamate neurotransmission (Portella *et al.* 2011; Haroon *et al.* 2017). However, we found no evidence of a negative correlation between duration of illness and glutamate levels. In addition, peripheral inflammation, as measured by CRP, did not correlate significantly with any of the MRS measures in the present study; indeed in the PUT, if anything, CRP tended to correlate negatively with glutamine levels. In addition, there was no correlation between glutamine concentration in the PUT and clinical measures of anhedonia or fatigue. However, it must be acknowledged that few of the patients in the current study had significantly raised levels of CRP. Also, although changes in peripheral inflammatory markers such as CRP have been commonly shown in depressed patients, how far these peripheral markers reflect central inflammatory changes is open to question (Setiawan *et al.* 2015).

Another recent investigation identified significantly high glutamate and glutamine levels in ACC in un-medicated depressed patients *v.* healthy controls, though peripheral inflammatory markers in these patients were not described (Abdallah *et al.* 2017). Interestingly, in the latter investigation, as in our study, there was a positive correlation between BDI score and glutamine but not the glutamate level in ACC. Taken together the data suggest that a variety of changes in glutamate and glutamine levels are present in depressed patients and further work is needed to understand the basis of this heterogeneity. In many previous studies, depressed patients were studied on antidepressant medication and while there is no clear link between antidepressant medication and lowered glutamate levels in anterior brain regions (Luykx *et al.* 2012; Arnone *et al.* 2015; Godlewska *et al.* 2015), it seems likely that the presence of antidepressant treatment in a depressed patient is a marker of treatment-resistance; this might be a relevant factor in determining glutamate status.

Synaptic glutamate release and glutamate–glutamine cycling are highly dynamic processes, which mean that interpretation of a change in a single, static measure of glutamine in depression must be tentative (Yüksel & Öngür, 2010; Bond & Lim, 2014). However, glutamine is largely found in glia where it is produced by the action of the enzyme, glutamine synthetase, on glutamate taken up from the synapse into glia (Zhou & Danbolt, 2014; Haroon *et al.* 2017). Indeed, this uptake of glutamate and its conversion to glutamine is one of the main mechanisms by which glutamate neurotoxicity is prevented, and is critically dependent on glial function. Thus the increased levels of glutamine found in the present study would not be straightforwardly compatible with the glial deficits that have been reported in neuropathological studies of depression (Cotter *et al.* 2001), and our findings are more consistent with an increased release and metabolism of glutamate in the PUT in depression.

The PUT has been implicated in the circuitry models of depression which emphasise the role of cortical–striatal projections in reward and motivational processes (Gunaydin & Kreitzer, 2016). Indeed, much of the glutamate neurotransmission in the PUT is derived from descending cortical fibres (Greenamyre, 2001). A recent meta-analysis of resting state fMRI studies in un-medicated depressed patients found altered activity in fronto-limbic systems, particularly implicating dorsolateral PFC and its connectivity to the PUT (Zhong *et al.* 2016). The authors suggested that altered activity in the PUT might be associated with deficits in social emotional regulation consistent with recent work that shows a role for caudate and PUT in emotional reappraisal (Ochsner *et al.* 2012). Of course, the PUT has a long-recognised role in motor behaviour (Gunaydin & Kreitzer, 2016) and in this respect, it is of interest that increased dopamine D2 receptor binding found in some studies of the PUT in depressed patients may correlate with the extent of psychomotor retardation (Meyer *et al.* 2006). There are important interactions between dopamine and glutamate release in the striatum which may also be relevant to the present findings (Gunaydin & Kreitzer, 2016).

While our study has a number of strengths, such as the inclusion of a large number of un-medicated depressed patients and advanced 7 T imaging, the finding of increased glutamine levels in the PUT was not hypothesised and will require confirmation in a further investigation with bilateral imaging of the PUT. In view of the potentially important role of inflammation in the pathophysiology of depression and glutamate neurotransmission (Haroon *et al.* 2016; 2017), future studies should also include a subgroup of depressed patients with clearer evidence of peripheral inflammation. However, the current study does suggest altered glutamatergic activity in the PUT in patients with major depression. This abnormality, if it can be confirmed, is likely to reflect the role of the striatum, including the PUT, in the cortical–striatal circuitry that regulates the emotional and reward-driven behaviours that underpin the clinical symptomatology of depressive states. We suggest that future MRS investigations in depression should include studies of basal ganglia as well as the more usually studied, cortical brain regions.

## Supplementary material

For supplementary material accompanying this paper visit https://doi.org/10.1017/S0033291717003373. click here to view supplementary material
