# Supplementary material for: Brain glutamate in medication-free depressed patients: a proton MRS study at 7 Tesla
Source: Psychol Med. 2017 Dec 11;48(10):1731–7. doi: 10.1017/S0033291717003373 (PMC6088784; doi:10.1017/S0033291717003373)
Supplement: Supplementary file 1 [file S0033291717003373sup.zip › S0033291717003373sup002.docx]

Supplementary Table 1. Mean (SEM) measures of spectral quality. SNR= signal to noise ratio; FWHM= full-width at half maximum; ACC – anterior cingulate cortex, OCC – occipital cortex, PUT – putamen.

|  | Patients with MDD | Healthy controls |
| --- | --- | --- |
| SNR (ACC) | 41.4 (1.0) | 43.9 (1.1) |
| SNR (OCC) | 38.7 (1.02) | 38.23 (1.09) |
| SNR (PUT) | 18.82 (0.60) | 18.58 (0.62) |
| FWHM (ACC) (Hz) | 11.3 (0.29) | 10.9 (0.29) |
| FWHM (OCC) (Hz) | 10.4 (0.29) | 11.1 (0.29) |
| FWHM (PUT) (Hz) | 13.9 (0.29) | 14.9 (0.59) |
