# Supplementary figures and images for: Brain glutamate in medication-free depressed patients: a proton MRS study at 7 Tesla
Source: Psychol Med. 2017 Dec 11;48(10):1731–7. doi: 10.1017/S0033291717003373 (PMC6088784; doi:10.1017/S0033291717003373)

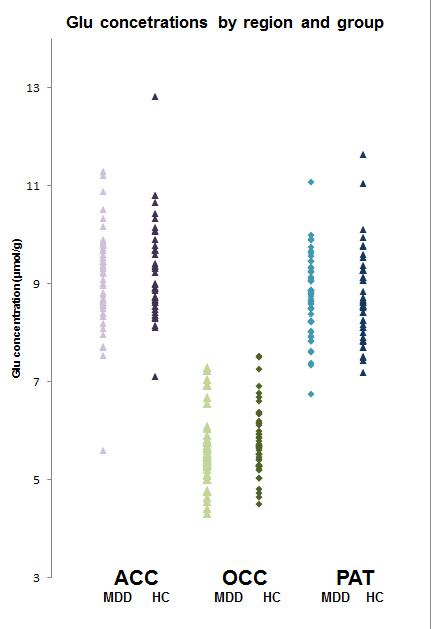

Supplement: Supplementary file 1 [file S0033291717003373sup.zip › S0033291717003373sup001.jpg]
